# Supplementary material for: Baseline Characterization of the Gut Microbiota of Field and Colony Populations of Phlebotomus tobbi and Preliminary Assessment of the Anti-Leishmanial Activity of Cultivable Bacteria
Source: Pathogens. 2026 Jun 23;15(7):658. doi: 10.3390/pathogens15070658 (PMC13416292; doi:10.3390/pathogens15070658)
Supplement: Supplementary file 1 [file pathogens-15-00658-s001.zip › pathogens-4357168-File S1/DADA2_ASV_Distribution/Read_Processing_Summary.html]

| internal\_id | customer\_label | rawseqs(R1+R2) | trimmed\_seqs(R1+R2) | dada2\_infered | chimera\_seqs | chimera\_free\_seqs | unique\_seqs | seqs(after\_size\_filtration) | final\_unique\_seqs |
| --- | --- | --- | --- | --- | --- | --- | --- | --- | --- |
| zr4724\_1V3V4 | Patient1 | 79232 | 77392 | 38154 | 9323 | 28831 | 37 | 28796 | 32 |
| zr4724\_2V3V4 | Patient2 | 97662 | 95912 | 47289 | 8819 | 38470 | 20 | 38463 | 19 |
| zr4724\_3V3V4 | Patient3 | 83522 | 81642 | 39820 | 10144 | 29676 | 23 | 29666 | 22 |
| zr4724\_4V3V4 | Patient4 | 87032 | 85500 | 41745 | 11112 | 30633 | 32 | 30584 | 24 |
| zr4724\_5V3V4 | Patient5 | 99216 | 97194 | 47770 | 9974 | 37796 | 27 | 37781 | 23 |
| zr4724\_6V3V4 | Patient6 | 79860 | 78414 | 38956 | 685 | 38271 | 23 | 38215 | 16 |
| zr4724\_7V3V4 | Patient7 | 97082 | 95394 | 47144 | 9409 | 37735 | 34 | 37676 | 24 |
| zr4724\_8V3V4 | Patient8 | 102790 | 100800 | 49854 | 13441 | 36413 | 35 | 36330 | 22 |
| zr4724\_9V3V4 | Patient9 | 59902 | 58722 | 28599 | 7172 | 21427 | 32 | 21410 | 28 |
| zr4724\_10V3V4 | Patient10 | 60478 | 59340 | 29418 | 3133 | 26285 | 24 | 26251 | 18 |
